# Influenza A Viruses of Human Origin in Swine, Brazil

**DOI:** 10.3201/eid2108.141891

**Published:** 2015-08

**Authors:** Martha I. Nelson, Rejane Schaefer, Danielle Gava, Maurício Egídio Cantão, Janice Reis Ciacci-Zanella

**Affiliations:** Fogarty International Center of the National Institutes of Health, Bethesda, Maryland, USA (M.I. Nelson);; EMBRAPA Swine and Poultry, Concordia, Brazil (R. Schaefer, D. Gava, M.E. Cantão, J.R. Ciacci-Zanella)

**Keywords:** viruses, influenza A virus, swine, pigs, Brazil, evolution, interspecies transmission, reassortment, humans, influenza, zoonoses

## Abstract

The evolutionary origins of the influenza A(H1N1)pdm09 virus that caused the first outbreak of the 2009 pandemic in Mexico remain unclear, highlighting the lack of swine surveillance in Latin American countries. Although Brazil has one of the largest swine populations in the world, influenza was not thought to be endemic in Brazil’s swine until the major outbreaks of influenza A(H1N1)pdm09 in 2009. Through phylogenetic analysis of whole-genome sequences of influenza viruses of the H1N1, H1N2, and H3N2 subtypes collected in swine in Brazil during 2009–2012, we identified multiple previously uncharacterized influenza viruses of human seasonal H1N2 and H3N2 virus origin that have circulated undetected in swine for more than a decade. Viral diversity has further increased in Brazil through reassortment between co-circulating viruses, including A(H1N1)pdm09. The circulation of multiple divergent hemagglutinin lineages challenges the design of effective cross-protective vaccines and highlights the need for additional surveillance.

## Abstract 2

Multiple divergent lineages challenge the design of cross-protective vaccines and highlight the need for additional surveillance.

Influenza A viruses circulating in swine (swIAVs) are of major economic concern for the swine industry and a pandemic threat for humans. The H1N1 influenza pandemic of 2009 was associated with a virus of swine origins (*1*) that caused its first outbreak in humans in Mexico in early 2009 (*2*). However, the evolutionary origins of influenza A(H1N1)pdm09 (pH1N1) virus in swine are poorly understood, and no potential progenitor viruses have been detected in swine in any part of the world. The relatively small number of swIAVs that have been characterized in Latin America make it particularly difficult to confirm or negate the possibility of pH1N1 evolving in swine in Mexico or another Latin American country before emergence in humans.

Since 2009, transmission of pH1N1 virus from humans to pigs has been documented in numerous countries spanning 6 continents (*3*–*13*), including many countries where influenza viruses previously had not been detected in swine, such as Australia (*14*), Finland (*15*), and Cameroon (*16*). pH1N1 virus has been identified in swine in several Latin American countries, including Argentina (*17*), Brazil (*18*,*19*), Colombia (*20*), and Mexico (*21*), because of human-to-swine transmission events that have occurred since 2009. In Argentina, multiple subtypes of viruses of human seasonal virus origin also have been identified in swine (*22*). Although Brazil hosts one of the largest swine populations in the world (≈41 million hogs), little evidence existed of swIAV circulation in swine herds in Brazil before 2009 (*23*–*25*). Influenza virus in pigs was first detected in Brazil in 1974, and the isolated virus was closely related to the classical North American swine virus A/swine/Illinois/1/63/H1N1 (*24*). However, relatively little clinical illness was observed in pigs in Brazil until 2009. Since 2009, Brazil’s swine population has experienced outbreaks of pH1N1 that are associated with respiratory illness. These outbreaks have been located primarily in the country’s major swine production regions in southern, midwestern, and southeastern Brazil (Figure 1). As a result, surveillance efforts for IAVs in swine populations in Brazil have increased, revealing additional influenza virus diversity. Serum collected from swine in southeastern Brazil during January–March 2009 indicated the widespread presence of antibodies cross-reactive to multiple antigenically distinct subtypes in swine: North American classical swine H1N1 (44.4%), North American triple-reassortant swine H3N2 (23.5%), and human-like H1N1 (38.3%) (*25*). In southern Brazil, the seroprevalence of the H3N2 subtype was recently found to be ≈20% (*23*). A human-like H1N2 virus was isolated from captive wild boars (*26*) and from swine (*27*) in Brazil.

**Figure 1:**
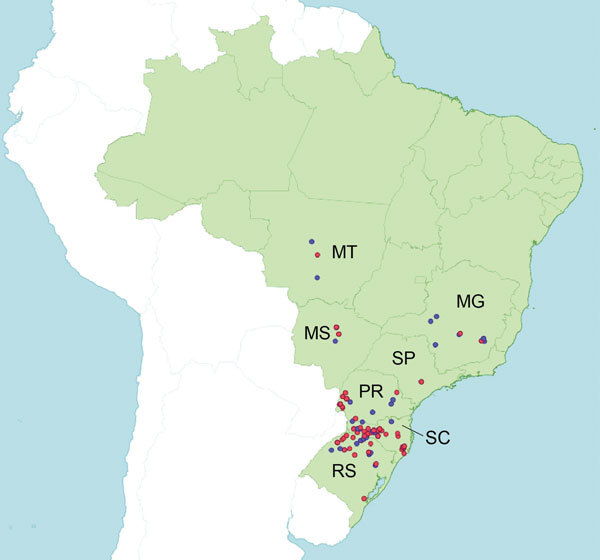
Areas of swine production in 7 states in southern, midwestern, and southeastern Brazil: Rio Grande do Sul (RS) State (total swine population ≈7.0 million), Santa Catarina (SC) State (≈9.0 million swine), Paraná (PR) State (≈6.0 million swine), Mato Grosso (MT) State (≈2.4 million swine), Mato Grosso do Sul (MS) State (≈1.3 million swine), Minas Gerais (MG) State (≈5.4 million swine), and São Paulo (SP) State (≈1.8 million swine). Red dots indicate pig farms sampled where at least 1 sample was positive for influenza by PCR; blue dots indicate farms where all samples were negative for influenza.

Here, a phylogenetic analysis of newly sequenced influenza viruses from Brazil’s swine herds provides evidence that multiple IAVs of human seasonal virus origin have been circulating in swine for more than a decade. These particular H3N2 and H1N2 swIAV clades appear to be specific to Brazil. The co-circulation of multiple genetically diverse swIAV lineages of the H1N1, H1N2, and H3N2 subtypes introduces new challenges for the control of influenza in Brazil’s swine herds, including development of cross-protective vaccines.

## Methods

### Data Collection and Sequencing

A total of 1,881 nasal swab and 89 lung tissue samples were collected from swine from 131 pig farms in southern, midwestern, and southeastern Brazil during 2009–2012 (Figure 1). Nursery and growing pigs were selected for nasal swab sampling during 2 cross-sectional studies in 2010 and 2011, where pigs showing typical clinical signs of influenza infection (e.g., fever, labored abdominal breathing, and dyspnea) were preferentially chosen for sampling. Lung samples were collected from pigs of different production phases (suckling, nursery, and fattening) and submitted to a private diagnostic laboratory for necropsy and/or histopathologic examination, and positive lung samples for IAV by immunohistochemical analysis were sent to the virology laboratory at EMBRAPA (Brazilian Agricultural Research Corporation, Concordia, Brazil) for virus isolation, reverse transcription quantitative PCR (RT-qPCR), and sequencing.

IAV was detected from nasal swab samples from pigs on 24 of 62 farms. Fifty-nine (3.13%) of 1,881 nasal swab samples analyzed were positive by RT-qPCR. Moreover, 58 (65.16%) of 89 lung samples with pneumonic lesions, from 44 pig farms, were positive for IAV by RT-qPCR. Viral RNA was extracted from the original samples and tested for the matrix gene of IAV and pH1N1 IAV by 2 separate RT-qPCRs (*28*,*29*). Virus was isolated by inoculating lung tissue supernatant or nasal swab samples into MDCK cells or into specific pathogen–free embryonated chicken eggs (*30*). To confirm virus isolation, we tested the supernatants or allantoic fluids for hemagglutinin (HA) activity and by RT-qPCR. Nasal swab and lung samples were considered negative for IAV if a second passage in specific pathogen–free chicken eggs or in MDCK cells was negative by HA test and RT-PCR. Of the 117 PCR-positive samples, 41 virus isolates were obtained: 14 from nasal swab samples and 27 from lung samples.

We conducted whole-genome sequencing using an ABI 3130xl analyzer (Applied Biosystems, Foster City, CA, USA) and Illumina MiSeq (Illumina, San Diego, CA, USA). PCR was conducted by using a set of primers specific for the amplification of the 8 IAV gene segments (*31*,*32*) and a primer set for the amplification of pH1N1 gene segments. For sequencing on an Illumina MiSeq, RT reaction and amplification of the 8 IAV gene segments were conducted by using primers as previously described (*32*,*33*). In total, at least partial sequence data were obtained for 35 IAVs. Of these, 16 swIAVs provided whole-genome sequence data of sufficient quality to be included in the final phylogenetic analysis.

### Phylogenetic Analysis

Nucleotide alignments were generated for 11 discrete datasets: H1 (human seasonal virus–like, 209 viruses), H1 (pandemic virus–like, 451 viruses), H3 (463 viruses), N1 (pandemic virus–like, 311 viruses), N2 (683 viruses), and the 6 internal gene segments (polymerase basic [PB] 2, PB1, polymerase acidic [PA], nucleoprotein [NP], matrix [MP], and nonstructural [NS]). Each dataset comprised 1) Brazilian swIAVs sequenced for this study; 2) Brazilian swIAVs that we sequenced and published previously (*26*,*27*); and 3) related human and swine viruses, collected globally, that were downloaded from the Influenza Virus Resource (*34*) available in GenBank, which were studied previously (*35*) (Table 1; Technical Appendix Table). To accommodate relatively long but not fully complete sequences, the H3 alignment was trimmed to 1158 nt and the N2 alignment was trimmed to 1161 nt, and sequences less than this length were excluded.

**Table 1:** Characteristics and phylogenetic position of 16 influenza A viruses from swine sequenced at EMBRAPA (Brazilian Agricultural Research Corporation)*

Virus	State†	Date collected	Specimen type	Isolation method	Segments			
					HA‡	NA§	Matrix	Other internal¶
A/swine/Brazil/185-11-7/2011/H1N2	SC	2011 Jul 6	NS	ECE/MDCK	H1s	N2-a	pdm	pdm
A/swine/Brazil/232-11-13/2011/H1N2	SC	2011 Aug 17	NS	ECE	H1s	N2-a	pdm	pdm
A/swine/Brazil/232-11-14/2011/H1N2	SC	2011 Aug 17	NS	ECE	–	N2-a	pdm	–
A/swine/Brazil/31-11-1/2011/H1N2	PR	2011 Feb 28	Lung	ECE	H1s	N2-a	pdm	pdm
A/swine/Brazil/31-11-3/2011/H1N2	PR	2011 Feb 28	Lung	ECE	H1s	N2-b	pdm	pdm
A/wild boar/Brazil/214-11-13D/2011/H1N2	RS	2011 Jul 25	Lung	#	H1s	N2-b	pdm	pdm
A/swine/Brazil/231-11-1/2011/H3N2	SC	2011 Aug 17	NS	ECE/MDCK	H3	–	pdm	–
A/swine/Brazil/355-11-6/2011/H3N2	RS	2011 Oct 27	NS	ECE	H3	N2-a	pdm	–
A/swine/Brazil/365-11-6/2011/H3N2	MS	2011 Nov 10	NS	ECE/MDCK	H3	–	pdm	–
A/swine/Brazil/365-11-7/2011/H3N2	MS	2011 Nov 10	NS	MDCK	H3	N2-a	pdm	pdm
A/swine/Brazil/12A/2010/H1N1	SC	2010 Jan 30	Lung	ECE	H1p	N1p	pdm	–
A/swine/Brazil/18/2012/H1N1	RS	2012 Feb 10	Lung	ECE	H1p	N1p	pdm	pdm
A/swine/Brazil/66/2011/H1N1	SC	2011 Apr 13	Lung	ECE	H1p	N1p	pdm	pdm
A/swine/Brazil/107/2010/H1N1	SC	2010 Jul 15	NS	ECE	H1p	N1p	pdm	pdm
A/swine/Brazil/132/2009/H1N1	SC	2009 Sep 9	Lung	ECE	H1p	–	pdm	–
A/swine/Brazil/263/2012/H1N1	SC	2012 Nov 27	Lung	ECE	H1p	N1p	pdm	pdm

**Figure 2:**
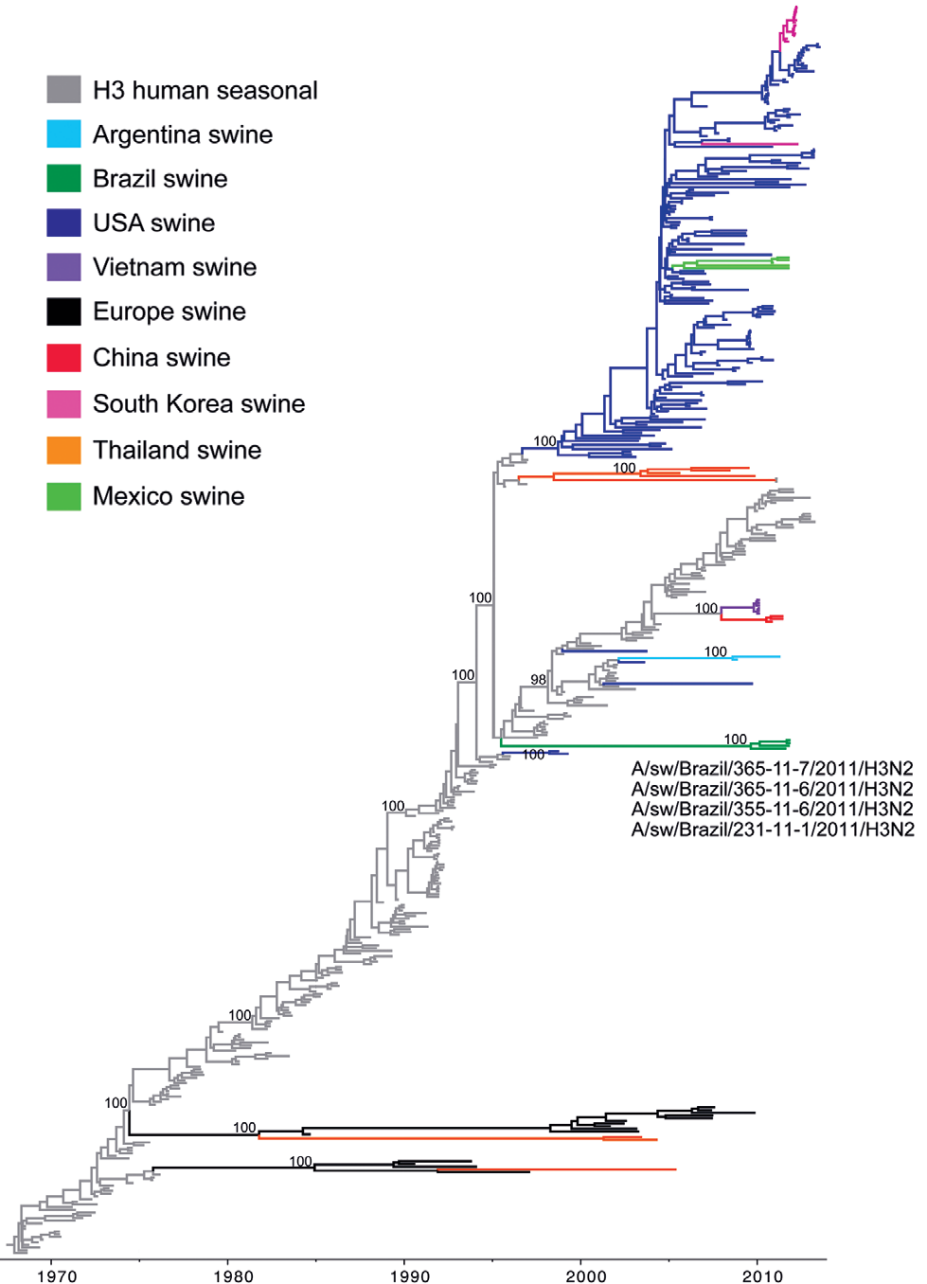
Phylogenetic relationships between human and swine influenza H3 segments. Time-scaled Bayesian maximum clade credibility (MCC) tree inferred for the hemagglutinin (H3) sequences of 463 viruses, including 4 viruses sequenced for this study from swine in Brazil, A/swine/Brazil/365-11-7/2011(H3N2), A/swine/Brazil/231-11-1/2011(H3N2), A/swine/Brazil/355-11-6/2011(H3N2), and A/swine/Brazil/365-11-6/2011(H3N2); 251 human seasonal H3 viruses collected globally during 1968–2013; and 208 closely related swine viruses collected globally that have been studied previously (*35*). Gray indicates branches of human seasonal influenza A(H3N2) virus origin. Branches associated with viruses from swine are shaded by country/area of origin: light blue, Argentina; dark green, Brazil; dark blue, United States; purple, Vietnam; black, Europe; red, China; pink, Korea; orange, Thailand; light green, Mexico. Posterior probabilities >0.9 are included for key nodes.

**Figure 3:**
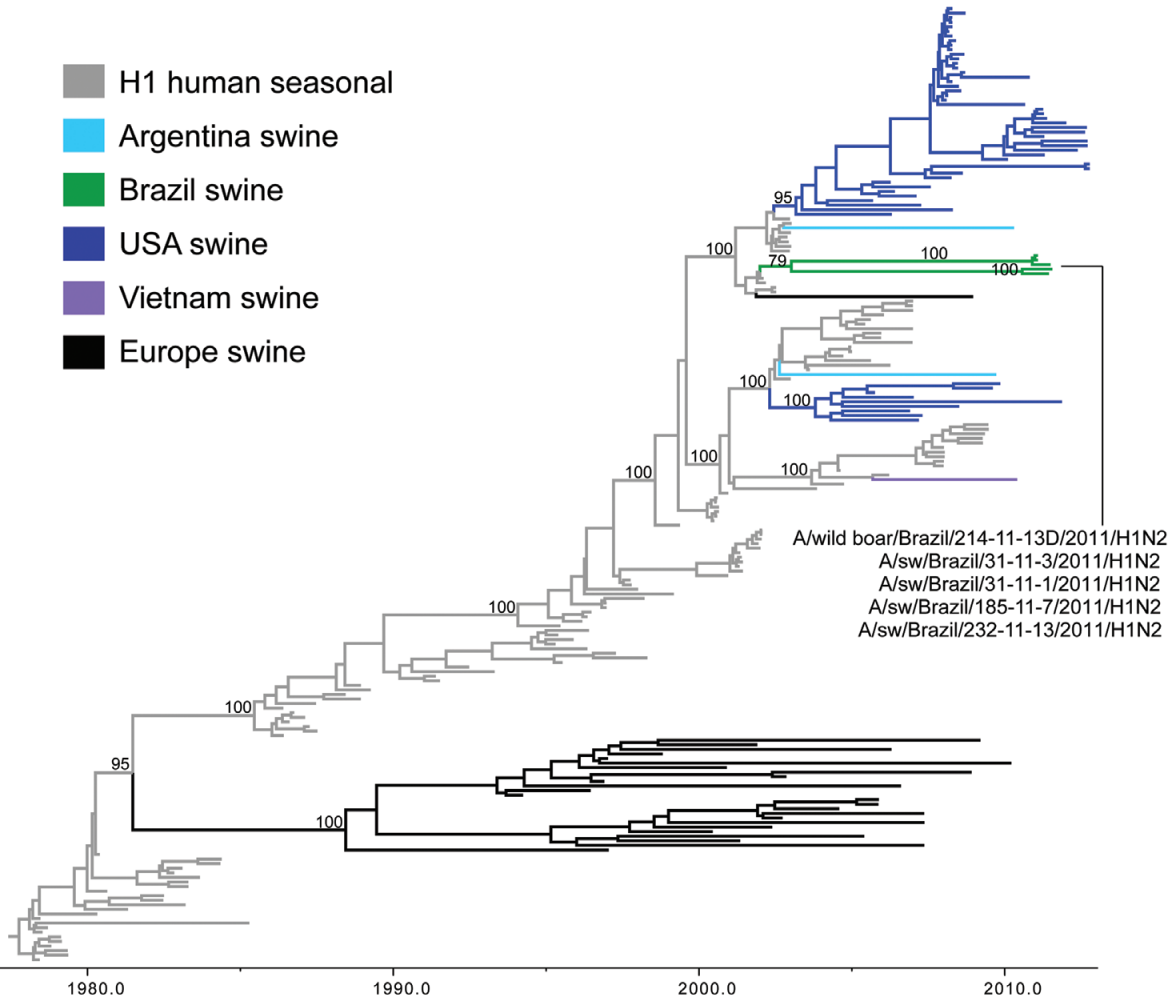
Phylogenetic relationships between human and swine influenza H1 segments. Time-scaled Bayesian maximum clade credibility (MCC) tree inferred for the hemagglutinin (H1) sequences of 209 viruses, including 2 swine viruses from Brazil sequenced for this study, A/swine/Brazil/185-11-7/2011(H1N2) and A/swine/Brazil/232-11-13/2011(H1N2); 3 swine influenza A viruses from Brazil sequenced previously, A/wild boar/Brazil/214-11-13D/2011(H1N2) (*26*), A/swine/Brazil/31-11-1/2011(H1N2), and A/swine/Brazil/31-11-3/2011(H1N2) (*27*); 120 human seasonal H1 viruses collected globally during 1978–2008; and 84 closely related swine viruses collected globally that have been studied previously (*35*). Gray indicates branches of human seasonal influenza A(H3N2) virus origin. Branches associated with viruses from swine are shaded by country/area of origin: light blue, Argentina; dark green, Brazil; dark blue, United States; purple, Vietnam; black, Europe; red, China; pink, Korea; orange, Thailand; light green, Mexico.

**Figure 4:**
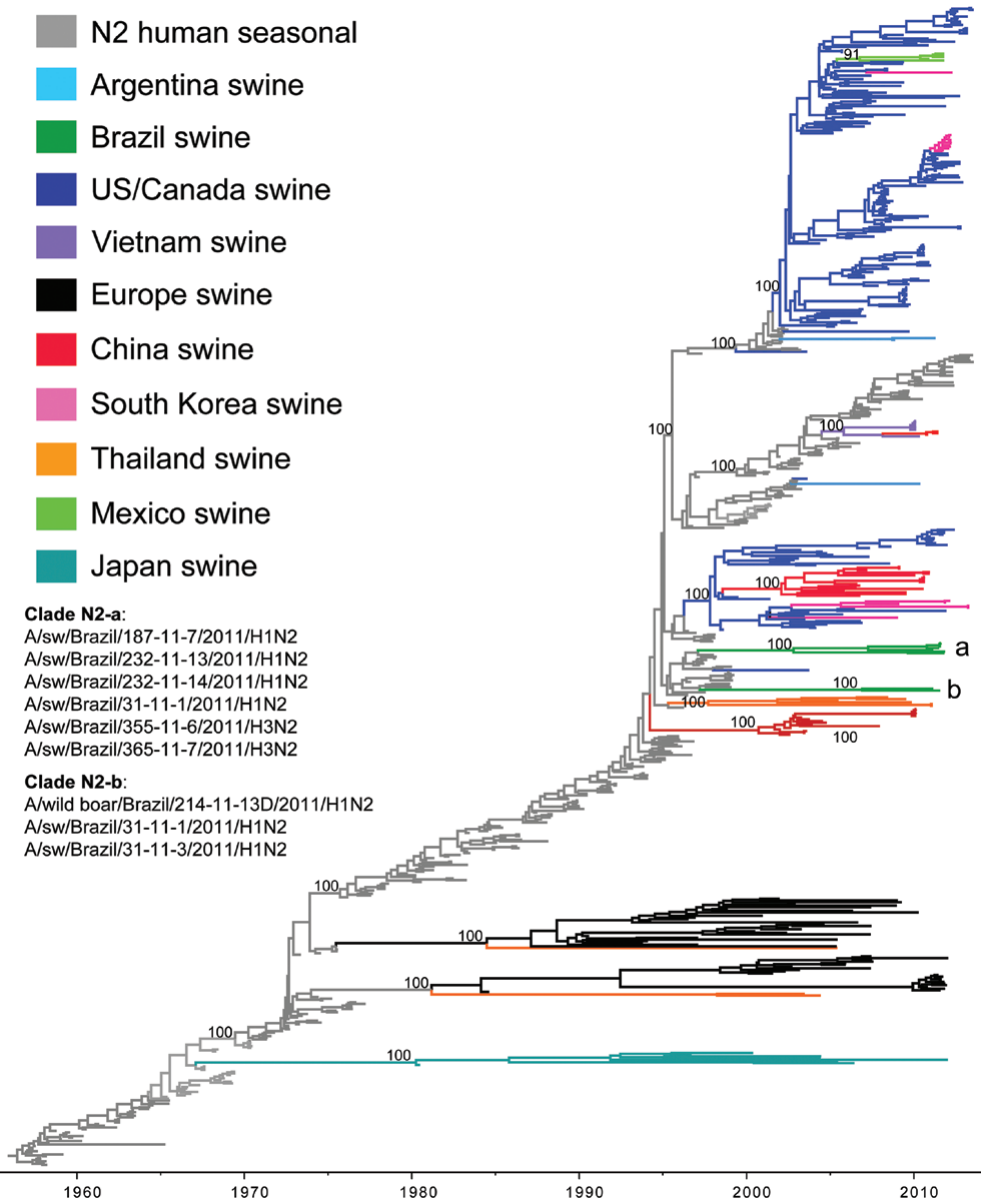
Phylogenetic relationships between human and swine influenza N2 segments. Time-scaled Bayesian maximum clade credibility (MCC) tree inferred for the neuraminidase (N2) sequences of 682 viruses, including 6 swine viruses from Brazil sequenced for this study, A/swine/Brazil/185-11-7/2011(H1N2), A/swine/Brazil/232-11-13/2011(H1N2), A/swine/Brazil/232-11-14/2011(H1N2), A/swine/Brazil/31-11-1/2011(H1N2), A/swine/Brazil/355-11-6/2011(H3N2), and A/swine/Brazil/365-11-7/2011(H3N2); 2 swine influenza A viruses from Brazil sequenced previously, A/wild boar/Brazil/214-11-13D/2011(H1N2) (*26*) and A/swine/Brazil/31-11-3/2011(H1N2) (*27*); 325 human seasonal N2 viruses collected globally during 1957–2013; and 350 closely related swine viruses collected globally that have been studied previously (*35*). Gray shading indicates branches of human seasonal influenza A(H3N2) virus origin. Branches associated with viruses from swine are shaded by country/area of origin: light blue, Argentina; dark green, Brazil; dark blue, United States; purple, Vietnam; black, Europe; red, China; pink, Korea; orange, Thailand; light green, Mexico; turquoise, Japanese swine viruses. Posterior probabilities >0.9 are included for key nodes, and the 2 clades of Brazilian swine viruses are labeled a and b (Table 1).

*ECE, embryonated chicken egg; HA, hemagglutinin; MS, Mato Grosso do Sul State; NA, neuraminidase; NS, nasal swab; pdm, pandemic; PR, Parana State; RS, Rio Grande do Sul State; SC, Santa Catarina State; –, insufficient sequence data available for analysis. †Location of virus collection among 4 states in southern and midwestern regions Brazil provided for each sample (Figure 1). ‡Viruses are classified as related to human seasonal H1 (H1s, Figure 3), human seasonal H3 (Figure 2), or pandemic H1 (H1p) (Technical Appendix Figure 4). §Viruses are classified as pandemic (N1p; Technical Appendix Figure 5) or 1 of the 2 clades of human seasonal N2 (a and b; Figure 4). ¶Polymerase basic 2, polymerase basic 1, polymerase acidic, nucleoprotein, and nonstructural. #Sequencing was performed directly from the original lung sample for A/wild boar/Brazil/214-11-13D/2011/H1N2.

For each of the 5 sets of sequence data, an alignment was generated by using MUSCLE v3.8.31 (*36*), with manual correction using the program Se-Al (http://tree.bio.ed.ac.uk/software/seal/). The phylogenetic relationships of each of the 5 datasets were inferred by using the maximum-likelihood (ML) method available in RAxML v7.2.6 (*37*), incorporating a general time-reversible model of nucleotide substitution with a γ-distributed rate variation among sites. To assess the robustness of each node, we conducted a bootstrap resampling process (500 replicates), again using the ML method available in RAxML v7.2.6. Because of the computational complexity of these processes, we used the high-performance computational capabilities of the Biowulf Linux cluster at the National Institutes of Health (http://biowulf.nih.gov).

### Divergence Times

A time-scaled Bayesian approach was used to estimate the timing of the human-to-swine transmission events associated with the H1, H3, and N2 segments, due to the long branch lengths separating the Brazilian swine viruses from the most closely related human seasonal viruses. We used a relaxed uncorrelated lognormal molecular clock, a flexible Bayesian skyline plot (BSP) demographic model (10 piece-wise constant groups), and a general-time reversible model of nucleotide substitution with a γ-distributed rate variation among sites. Markov chain Monte Carlo was run separately 3 times for each dataset for at least 100 million iterations, with subsampling every 10,000 iterations. The analysis used BEAST v1.8.0 (*38*) and the BEAGLE library (*39*) to improve computational performance. All parameters reached convergence, as assessed visually by using Tracer v1.6 (http://tree.bio.ed.ac.uk/software/tracer/), with statistical uncertainty reflected by values of the 95% highest posterior density (HPD). The initial 10% of the chain was removed as burn-in, runs were combined by using LogCombiner v1.8.0 (http://beast.bio.ed.ac.uk/logcombiner), and maximum clade credibility (MCC) trees were summarized by using TreeAnnotator v1.8.0 (http://beast.bio.ed.ac.uk/treeannotator).

## Results

Among the newly sequenced swIAVs from Brazil, we identified multiple previously uncharacterized clades of viruses that are most closely related to human seasonal H3N2 and H1N2 viruses that circulated in humans during the 1990s and early 2000s, respectively (Figures 2–4). All of these viruses had matrix segments of pH1N1 origin, and all other internal genes that could be sequenced were also of pH1N1 origin (Table 1).

### Identification of New Human-Origin H3N2 IAVs in Swine

On the H3 phylogeny, the 4 H3N2 viruses from swine in Brazil in 2011 are monophyletic, forming a single clade supported by 100% posterior probability (Figure 2) and 100% bootstrap support (Technical Appendix Figure 1): A/swine/Brazil/365-11-7/2011(H3N2), A/swine/Brazil/231-11-1/2011(H3N2), A/swine/Brazil/355-11-6/2011(H3N2), and A/swine/Brazil/365-11-6/2011(H3N2) (Table 1; Technical Appendix Table). This clade is phylogenetically distinct from other swIAVs found in Argentina and North America that also are of human seasonal H3N2 virus origin and appears to be a previously uncharacterized introduction of a human seasonal H3N2 virus into swine (Figure 2; Technical Appendix Figure 1). The human H3N2 viruses that are most closely related to the Brazilian swIAVs were isolated during the late 1990s (e.g., A/Malaysia/13241/1997[H3N2]) and have a long branch length separating the clade of swIAVs from Brazil from the older human virus diversity.

The time-scaled MCC tree (Figure 2) indicates that human-to-swine transmission could have occurred at any time along this long branch between the node representing the estimated time to the most recent common ancestor of the clade of swIAVs from Brazil (2009.6; 95% HPD 2008.5–2010.4) and the node representing the time to the most recent common ancestor for the swine clade and most closely related human virus (1995.5; 95% HPD 1995.1–1995.9). The much higher intensity of sampling of human viruses than swine viruses means that the long branch length is more likely to represent unsampled swine viruses than unsampled human viruses and the actual time of human-to-swine introduction is likely to be closer to 1995.5. Detection of H3N2 swIAVs in 3 Brazilian states (Santa Catarina, Rio Grande do Sul, and Mato Grosso do Sul; Figures 1, 2) is consistent with extensive circulation of this swIAV within swine populations in southern and midwestern Brazil.

### Identification of New Human-Origin H1N2 IAVs in Swine

On the H1 phylogeny, the 5 H1N2 viruses collected from swine in Brazil during 2011 also are monophyletic, although the clade is supported by just below the 70% bootstrap threshold on the ML tree (64% bootstrap support, Technical Appendix Figure 2) and 80% posterior probability threshold on the MCC tree (79%, Figure 3). The lower support for this clade appears to be related to the early divergence of these swIAVs into 2 well-supported subclades (100% posterior probabilities). One subclade comprises 3 swIAVs collected in Paraná and Rio Grande do Sul States: A/swine/Brazil/31-11-1/2011(H1N2), A/swine/Brazil/31-11-3/2011(H1N2), and A/wild boar/Brazil/214-11-13D/2011(H1N2) (Table 1; Figures 1, 3). The second subclade comprises 2 swIAVs collected in Santa Catarina State: A/swine/Brazil/185-11-7/2011(H1N2) and A/swine/Brazil/232-11-13/2011(H1N2). The presence of H1N2 viruses from domestic swine and a wild boar within the first subclade is consistent with viral transmission between wild and domestic swine in Brazil.

The estimated time of transmission of the human H1N2 viruses into swine is 2002.1–2003.1 (95% HPD 2001.8–2004.7), which is consistent with the period of global circulation of the unusual reassortant H1N2 virus in humans (2001–2003). The most closely related human virus was isolated in 2002: A/New York/417/2002/H1N2. Because of the low availability of human H1N2 sequences from this period and the low posterior probability (MCC tree) and bootstrap support (ML tree) for the clade of Brazilian H1N2 viruses, we cannot conclude with certainty that the H1N2 swIAVs identified in Brazil represent a single viral introduction from humans into pigs instead of 2 contemporaneous introductions involving similar human H1N2 viruses. The most parsimonious explanation, however, is that a single introduction of a human seasonal H1N2 virus into swine occurred, and shortly thereafter the virus diversified into 2 subclades that co-circulated in swine until at least 2011, when these H1N2 viruses were identified in swine in Brazil.

### Two Introductions of Human N2 Segments in Swine

In contrast to the H3 and H1 phylogenies (Figures 2, 3), the Brazilian swIAVs are not monophyletic on the N2 phylogeny, representing 2 different introductions of the N2 segment from human seasonal H3N2 viruses into swine in Brazil (Figure 4). One N2 clade (clade N2-a, Figure 4) is defined by 100% posterior probability support and 90% bootstrap support (Technical Appendix Figure 3) and comprises 6 Brazilian swIAVs: A/swine/Brazil/185-11-7/2011(H1N2), A/swine/Brazil/31-11-1/2011(H1N2), A/swine/Brazil/232-11-13/2011(H1N2), A/swine/Brazil/232-11-14/2011(H1N2), A/swine/Brazil/355-11-6/2011(H3N2), and A/swine/Brazil/365-11-7/2011(H3N2) (Table 1). Within the N2-a clade, the 4 H1N2 viruses cluster separately from the 2 H3N2 viruses as 2 genetically distinct subclades (90% and 100% bootstrap support, respectively; online Technical Appendix Figure 3). The 6 swIAVs in N2-a were collected from 4 Brazilian states: Santa Catarina, Rio Grande do Sul, Paraná, and Mato Grosso do Sul (Figure 1). The other N2 clade (clade N2-b, Figure 4) consists of 2 swIAVs from Brazil of the H1N2 subtype, defined by 100% posterior probability and 90% bootstrap support (Technical Appendix Figure 3): A/wild boar/Brazil/214-11-13D/2011(H1N2) and A/swine/Brazil/31-11-3/2011(H1N2). These swIAVs were collected in Paraná and Rio Grande do Sul States, consistent with dispersal within southern Brazil despite the low number of samples (Figure 1; Table 1).

Both the Brazilian N2-a and N2-b clades are closely related to human seasonal H3N2 viruses that circulated during the late 1990s (e.g., A/New York/521/1998[H3N2] for clade N2-a and A/New York/251/1998[H3N2] for clade N2-b) and therefore have similar estimated times of human-to-swine transmission on the MCC tree: 1997.1–2002.8 (95% HPD 1996.3–2005.6) for clade N2-a and 1997.2–2006.9 (95% HPD 1996.1–2009.0) for N2-b (Figure 4). Again, the higher intensity of influenza surveillance in humans than in swine suggests that the 2 human-to-swine transmission events associated with clades N2-a and N2-b are more likely to have occurred during late 1990s. These estimates overlap with the timing of the introduction of the H3 segment into swine in Brazil (Figure 2), and a parsimonious explanation is that the H3 clade and clade N2-a both represent the same introduction of human H3N2 viruses during the late 1990s, although additional sequencing is needed. In contrast, no evidence exists that the N2 of human H1N2 origin has continued to circulate in swine in Brazil beyond its initial introduction during the early 2000s. Rather, during the decade since the introduction of H1N2 viruses into the swine population, the H1N2 viruses have acquired through reassortment 2 different N2 segments of human H3N2 origin that circulated in swine in Brazil (N2-a and N2-b), representing 2 independent reassortment events between H1N2 and H3N2 viruses.

### Introductions of Human Pandemic H1N1 IAVs into Swine in Brazil

The 6 viruses of human pH1N1 origin that we sequenced from swine in Brazil were not monophyletic on the H1 or N1 tree, indicating that these viruses represent multiple separate human-to-swine introductions rather than clonal expansion of a single human pH1N1 introduction into pigs (Table 1; Technical Appendix Figures 4, 5). Additional pH1N1 HA and neuraminidase (NA) sequences were available in GenBank from swine in Brazil; these sequences also were not monophyletic or closely related to the pH1N1 viruses that were sequenced in our study, suggesting additional introductions of human pH1N1 viruses into swine herds in Brazil. The lower phylogenetic resolution of the recently emerged pH1N1 virus and large number of singleton viruses make precise estimation of the number of human-to-swine introductions difficult. As a lower-bound estimate based on monophyletic clusters supported by high bootstrap values (>70) on the pandemic H1, N1, and PB2 trees (Technical Appendix Figures 4–6), we conservatively estimate 8 introductions of pH1N1 from humans into swine in Brazil, including additional sequence data from GenBank (Table 2). Including singleton viruses and poorly supported clusters, as many as 15 putative human-to-swine introductions in Brazil were evident on the HA phylogeny (Technical Appendix Figure 4). Some of the long branch lengths separating singleton swine viruses from human viruses might represent onward transmission of a pH1N1 introduction in the swine population in Brazil, but additional sequencing is needed to confirm sustained circulation. Although the resolution of the phylogeny inferred for the matrix segment is even lower, because of the short sequence length, the swIAVs from Brazil also are not monophyletic on the matrix tree, consistent with multiple introductions of pH1N1 into swine in Brazil (Technical Appendix Figure 7). Phylogenies also suggest that human pH1N1 viruses have been transmitted multiple times to swine in other Latin America countries, including Argentina, Colombia, and Mexico (Table 2; Technical Appendix Figures 4–6). Only in Costa Rica was the pH1N1 population monophyletic with high bootstrap support that suggests a single human-to-swine introduction.

**Table 2:** Introductions of influenza A(H1N1)pdm09 virus from humans to swine in Latin America

Introduction	Viruses	Collection date	Bootstrap*
Brazil			
1	A/swine/Brazil/1/2009/H1N1; A/swine/Brazil/2/2009/H1N1; A/swine/Brazil/3/2009/H1N1	2009 Aug 11; 2009 Aug 11; 2009 Aug 11	75 (HA), 72 (NA)
2	A/swine/Brazil/5/2009/H1N1; A/swine/Brazil/6/2009/H1N1	2009 Aug 28; 2009 Aug 28	97 (HA)
3	A/swine/Brazil/8/2009/H1N1; A/swine/Brazil/9/2009/H1N1; A/swine/Brazil/10/2009/H1N1	2009 Oct 5; 2009 Oct 5; 2009 Oct 5	85 (HA), 85 (NA)
4	A/swine/Brazil/12/2009/H1N1; A/swine/Brazil/13/2009/H1N1	2009 Oct 25; 2009 Nov 10	97 (HA)
5	A/swine/Brazil/19/2010/H1N1; A/swine/Brazil/20/2010/H1N1	2010 Sep 9; 2010 Sep 9	100 (HA), 100 (NA)
6	A/swine/Brazil/107/2010/H1N1 (SC); A/wild boar/Brazil/214-11-13D/2011/H1N2 (RS)	2010 Jul 15; 2011 Jul 25	84 (PB2), 83 (PB1)
7	A/swine/Brazil/31-11-1/2011/H1N2 (PR); A/swine/Brazil/31-11-3/2011/H1N2 (PR)	2011 Feb 28; 2011 Feb 28	100 (PB2), 100 (PB1)
8	A/swine/Brazil/185-11-7/2011/H1N2 (SC); A/swine/Brazil/232-11-13/2011/H1N2 (SC)	2011 Jul 6; 2011 Aug 17	99 (PB2)
Argentina			
1	A/swine/Argentina/CIP051-A204/2012/H1N1; A/swine/Argentina/CIP051-A207/2012/H1N1	2012 Feb ; 2012 Feb	90 (HA)
2	A/swine/Argentina/CIP051/BsAs76/2009/H1N1; A/swine/Argentina/CIP051-A160/2011/H3N2	2009 Oct; 2011 Apr	100 (PB2), 86 (PB1)
Colombia			
1	A/swine/Colombia/1-01/2009/H1N1; A/swine/Colombia/1101/2009/H1N1	2009 Aug; 2009 Nov	85 (HA)
2	A/swine/Colombia/1/2011/H1N1; A/swine/Colombia/2/2011/H1N1	2011 Jun 16; 2011 Jun 16	100 (HA)
3	A/swine/Colombia/3/2011/H1N1; A/swine/Colombia/4/2011/H1N1	2011 Jul 28; 2011 Jul 28	100 (HA)
Costa Rica	A/swine/Costa Rica/000125-15/2010/H1N1; A/swine/Costa Rica/000125-3/2010/H1N1; A/swine/Costa Rica/000125-20/2010/H1N1; A/swine/Costa Rica/000125-19/2010/H1N1; A/swine/Costa Rica/000125-16/2010/H1N1 A/swine/Costa Rica/000125-14/2010/H1N1	2011 Nov 1; 2011 Nov 1; 2011 Nov 1; 2011 Nov 1; 2011 Nov 1	100 (HA), 100 (NA)
Mexico	A/swine/Mexico/SG1443/2010/H1N1; A/swine/Mexico/SG1450/2011/H1N1	2010 Oct 2; 2011 Nov 9	100 (HA), 100 (NA)

*Bootstrap support for clusters of viruses associated with introductions of pH1N1 virus from humans into swine in Latin America (Technical Appendix Figures 4–6). HA, hemagglutinin; NA, neuraminidase; PB, polymerase basic; pHN1, influenza A(H1N1)pdm09.

### Reassortment with pH1N1 Internal Genes

Several additional introductions of pH1N1 were evident only on the internal gene trees (e.g., PB2; Technical Appendix Figure 6) resulting from reassortment with HA and NA segments of human seasonal virus origin (Tables 1,2). In contrast to the genetic diversity of the HA and NA segments in swine in Brazil, the genetic diversity of the 6 internal gene segments in Brazil was restricted to pH1N1, at least for those for which sequence data were available (Table 1). This pattern indicates that the internal genes of pH1N1 viruses have reassorted with the HA and NA of other swIAVs of human seasonal virus origin circulating in Brazil.

## Discussion

Our recently initiated influenza virus surveillance in Brazil uncovered multiple new lineages of swIAVs that are related to seasonal influenza viruses that circulated in humans more than a decade ago. These swIAVs have not been detected in any other country, including the well-sampled swine populations of the United States, although surveillance for swIAVs is infrequent or lacking altogether in neighboring Latin America countries. Therefore, the possibility that these swIAVs have circulated, or even originated, in other Latin America countries that do not routinely conduct surveillance in swine cannot be excluded. At this time, however, the most parsimonious explanation based on the data available is that human seasonal viruses were introduced at least 3 times into swine in Brazil: H3N2 viruses were introduced twice into swine during the late 1990s, and a human seasonal H1N2 virus was introduced during the early 2000s. Viral segments from all 3 introductions have persisted in swine at least until 2011, the most recent date of sample collection. However, the internal gene segments were replaced in the intervening years by reassortment with pH1N1 viruses transmitted from humans to swine in Brazil multiple times since 2009.

These findings highlight the importance of human-to-swine transmission in the evolution of swIAV diversity in Brazil. The detection of multiple influenza viruses of human seasonal and pandemic origin circulating within Brazil’s large swine herds is entirely consistent with the frequency of human-to-swine transmission that recently has been highlighted on a global scale for seasonal (*35*) and for pandemic (*40*) influenza viruses. The swIAVs of human seasonal and pandemic origin also have been identified in Argentina in recent years (*17*,*22*). Our findings also match the globally observed pattern that new HA and NA segments of human seasonal virus origin persist at higher rates than the internal genes, which frequently are replaced through reassortment by cccirculating swine viruses, particularly pH1N1 (*35*).

Our data provided no evidence of importation of any swIAVs into Brazil from another country’s swine population. None of the triple reassortant, classical, or delta lineage influenza viruses that are prevalent in swine populations in the United States and Canada were detected in Brazil. However, it is possible that other swIAV lineages circulate in Brazil that our surveillance did not detect. Notably, our surveillance in Brazil did not identify either of the 2 swIAV lineages (North American triple reassortant or avian-like Eurasian viruses) that combined to create the pH1N1 virus associated with the 2009 pandemic. Consequently, the geographic origins of the pH1N1 virus in swine before its emergence in humans in 2009 remain unclear. Additional swIAV surveillance in unstudied and understudied regions in Latin America is greatly needed to understand the spatial origins and evolution of the swine virus that gave rise to the first pandemic outbreak in Mexico in early 2009.

The identification of swIAV diversity in Brazil, particularly in the HA and NA segments, has implications for the design of effective influenza vaccines for Brazil, which are in development. Although influenza viruses had been isolated from swine in Brazil from as early as 1974, influenza was not believed to be a major problem clinically for swine until the 2009 pandemic (*18*,*19*). Our findings suggest that H3N2 and H1N2 viruses could have circulated for many years in Brazil but were not associated with major clinical illness and have become pathogenic only since 2009, after reassortment with co-circulating pH1N1 virus internal genes. Given the relatively limited number of samples we collected compared with the large population of ≈41 million swine in Brazil, additional surveillance is critically needed to understand the diversity of swIAVs in circulation. Further surveillance is also necessary to select representative vaccine strains that will best protect against the diversity of swIAVs circulating in Brazil. Finally, further antigenic characterization is needed to assess the age-specific pandemic threat presented by these new swIAVs. The H3N2 and H1N2 viruses are most closely related to human seasonal IAVs that circulated during the 1990s and 2000s, and children may have particularly low preexisting immunity.

Technical Appendix GenBank accession numbers for 16 swine influenza viruses from Brazil; and maximum-likelihood trees of human and swine H3, H1, N2, pandemic H1, pandemic N1, pandemic PB2, and pandemic matrix gene segments.
