# Supplementary material for: Influenza A Viruses of Human Origin in Swine, Brazil
Source: Emerg Infect Dis. 2015 Aug;21(8):1339–47. doi: 10.3201/eid2108.141891 (PMC4517702; doi:10.3201/eid2108.141891)
Supplement: Technical Appendix — GenBank accession numbers for 16 swine influenza viruses from Brazil; and maximum-likelihood trees of human and swine H3, H1, N2, pandemic H1, pandemic N1, pandemic PB2, and pandemic matrix gene segments. [file 14-1891-Techapp-s1.pdf]

Article DOI: http://dx.doi.org/10.3201/eid2108.141891

Influenza A Viruses of Human Origin in Swine, Brazil

Technical Appendix

Technical Appendix Table. GenBank accession numbers for 16 swine influenza viruses from Brazil sequenced at EMBRAPA (Brazilian Agricultural Research Corporation)*

	GenBank accession no.								
Virus	Polymerase basic 2	Polymerase basic 1	Polymerase acidic	Hemagglutinin	Nucleoprotein	Neuraminidase	Matrix	Nonstructural	Reference
A/swine/Brazil/185-11-7/2011/H1N2	KM507517	KM507516	KM507518	KM507519	KM507520	KM507521	KM507522	KM507523	
A/swine/Brazil/232-11-13/2011/H1N2	KM507524	KM507525	KM507526	KM507530	KM507527	KM507531	KM507528	KM507529	
A/swine/Brazil/232-11-14/2011/H1N2				KM507532		KM507534	KM507533		
A/swine/Brazil/31-11-1/2011/H1N2	KF680293	KF680294	KF680295	KF680296	KF680297	KF680298	KF680299	KF680300	(*27*)
A/swine/Brazil/31-11-3/2011/H1N2	KF680286	KF680285	KF680287	KF680291	KF680288	KF680292	KF680289	KF680290	(*27*)
A/wild boar/Brazil/214-11-13D/2011/H1N2	KF572613	KF572614	KF572615	KF572616	KF572617	KF572618	KF572619	KF572620	(*26*)
A/swine/Brazil/231-11-1/2011/H3N2				KM507535		KM507537	KM507536		
A/swine/Brazil/355-11-6/2011/H3N2				KM507503		KM507505	KM507504		
A/swine/Brazil/365-11-6/2011/H3N2				KM507506			KM507507		
A/swine/Brazil/365-11-7/2011/H3N2	KM507508	KM507509	KM507510	KM507511	KM507512	KM507513	KM507514	KM507515	
A/swine/Brazil/12A/2010/H1N1	KM507538	KM507539	KM507540	JF421756	KM507541	KM507542	KM507543	KM507544	(*19*)
A/swine/Brazil/18/2012/H1N1	KM496984	KM496985	KM496986	KM496987	KM496988	KM496989	KM496990	KM496991	
A/swine/Brazil/66/2011/H1N1	KM496992	KM496993	KM496994	KM496995	KM496996	KM496997	KM496998	KM496999	
A/swine/Brazil/107/2010/H1N1	KF683611	KF683612	KF683613	KF683614	KF683615	KF683616	KF683617	KF683618	(*26*)
A/swine/Brazil/132/2009/H1N1				KM497008			KM497009		
A/swine/Brazil/263/2012/H1N1	KM497000	KM497001	KM497002	KM497003	KM497004	KM497005	KM497006	KM497007	

Supplementary Materials

Table S1. GenBank accession numbers for 16 swine influenza viruses from Brazil sequenced at Embrapa (Brazilian Agricultural Research Corporation).

**Virus**	**GenBank accession numbers**								**Ref**
	PB2	PB1	PA	HA	NP	NA	M	NS	
A/sw/Brazil/185-11-7/2011/H1N2	KM507517	KM507516	KM507518	KM507519	KM507520	KM507521	KM507522	KM507523	
A/sw/Brazil/232-11-13/2011/H1N2	KM507524	KM507525	KM507526	KM507530	KM507527	KM507531	KM507528	KM507529	
A/sw/Brazil/232-11-14/2011/H1N2				KM507532		KM507534	KM507533		
A/sw/Brazil/31-11-1/2011/H1N2	KF680293	KF680294	KF680295	KF680296	KF680297	KF680298	KF680299	KF680300	(27)
A/sw/Brazil/31-11-3/2011/H1N2	KF680286	KF680285	KF680287	KF680291	KF680288	KF680292	KF680289	KF680290	(27)
A/wb/Brazil/214-11-13D/2011/H1N2	KF572613	KF572614	KF572615	KF572616	KF572617	KF572618	KF572619	KF572620	(26)
A/sw/Brazil/231-11-1/2011/H3N2				KM507535		KM507537	KM507536		
A/sw/Brazil/355-11-6/2011/H3N2				KM507503		KM507505	KM507504		
A/sw/Brazil/365-11-6/2011/H3N2				KM507506			KM507507		
A/sw/Brazil/365-11-7/2011/H3N2	KM507508	KM507509	KM507510	KM507511	KM507512	KM507513	KM507514	KM507515	
A/sw/Brazil/12A/2010/H1N1	KM507538	KM507539	KM507540	JF421756	KM507541	KM507542	KM507543	KM507544	(19)
A/sw/Brazil/18/2012/H1N1	KM496984	KM496985	KM496986	KM496987	KM496988	KM496989	KM496990	KM496991	
A/sw/Brazil/66/2011/H1N1	KM496992	KM496993	KM496994	KM496995	KM496996	KM496997	KM496998	KM496999	
A/sw/Brazil/107/2010/H1N1	KF683611	KF683612	KF683613	KF683614	KF683615	KF683616	KF683617	KF683618	(26)
A/sw/Brazil/132/2009/H1N1				KM497008			KM497009		

Technical Appendix Figure 1. Maximum-likelihood (ML) tree of human and swine influenza H3 segments. Tree is similar to that in Figure 2 but inferred using ML methods. Branch lengths represent genetic distance and are drawn to scale. Bootstrap values for key nodes >75 are provided. Asterisk indicates the clade of Brazilian viruses.

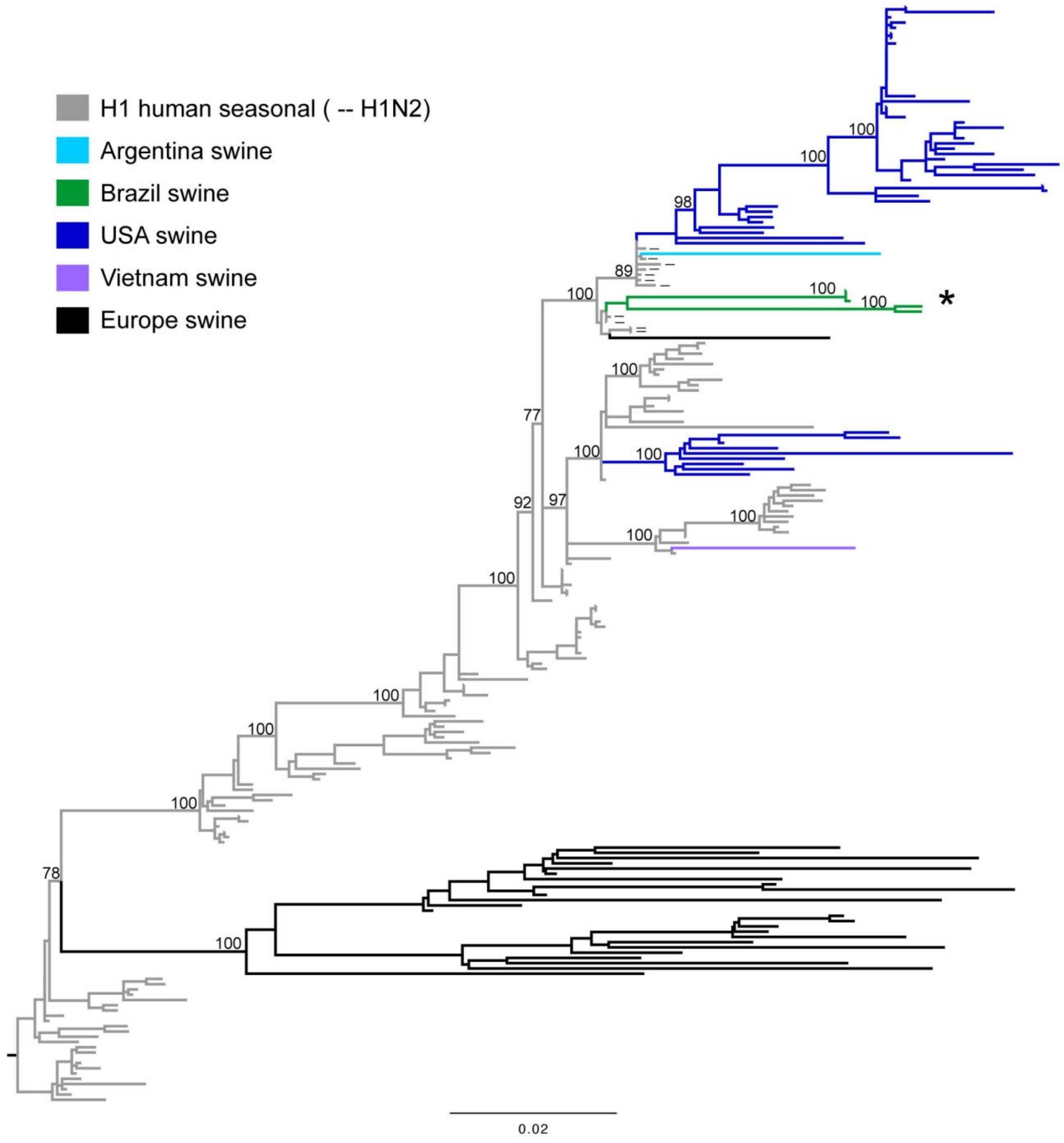

Technical Appendix Figure 3. Maximum-likelihood (ML) tree of human and swine influenza N2 segments. Tree is similar to that in Figure 4 but inferred using ML methods. Branch lengths represent genetic distance and are drawn to scale. Bootstrap values for key nodes >75 are provided. Asterisks indicate the 2 clades of Brazilian viruses.

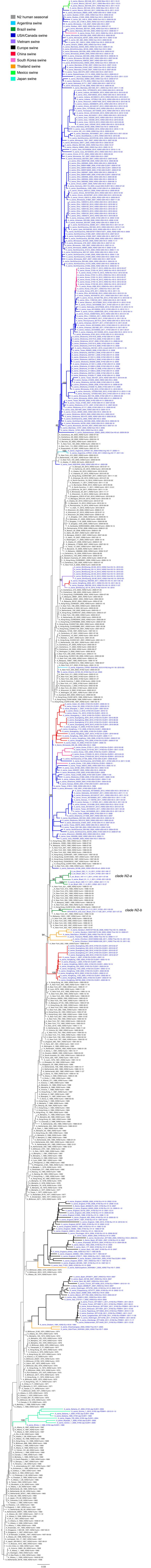

N2 human seasonal
Argentina swine
Brazil swine
USA/Canada swine
Vietnam swine
Europe swine
China swine
South Korea swine
Thailand swine
Mexico swine
Japan swine
clade N2-a
clade N2-b
0.05

Technical Appendix Figure 4. Maximum-likelihood (ML) tree of human and swine pandemic influenza H1 segments. Phylogenetic relationships of 451 pandemic H1 sequences are inferred using ML methods. Branch lengths represent genetic distance and are drawn to scale. Bootstrap values for key nodes >75 are provided. The H1p alignment comprises 4 Brazilian swine viruses that were sequenced for this study (A/swine/Brazil/18/2012/H1N1, A/swine/Brazil/66/2011/H1N1, A/swine/Brazil/263/2012/H1N1, and A/swine/Brazil/132/2009/H1N1); 2 Brazilian swine influenza A viruses sequenced previously (A/swine/Brazil/12A/2010/H1N1 [*19*] and A/swine/Brazil/107/2010/H1N1 [*26*); 401 human pandemic H1 sequences collected in Latin America during 2009–-2012; and 43 closely related H1 swine sequences collected in Latin America, including 12 from Brazilian swine that were studied previously (*18*). Swine viruses are shaded blue; swine viruses sequenced by EMPRAPA (Brazilian Agricultural Research Corporation) are shaded red. Solid shaded circles are shaded by country (green, Brazil; yellow, Colombia; black, Mexico; red, Costa Rica; blue, Argentina) represent putative viral introductions into swine. Viral introductions into swine that are supported by bootstrap values >75 are numbered in accordance with Table 3.

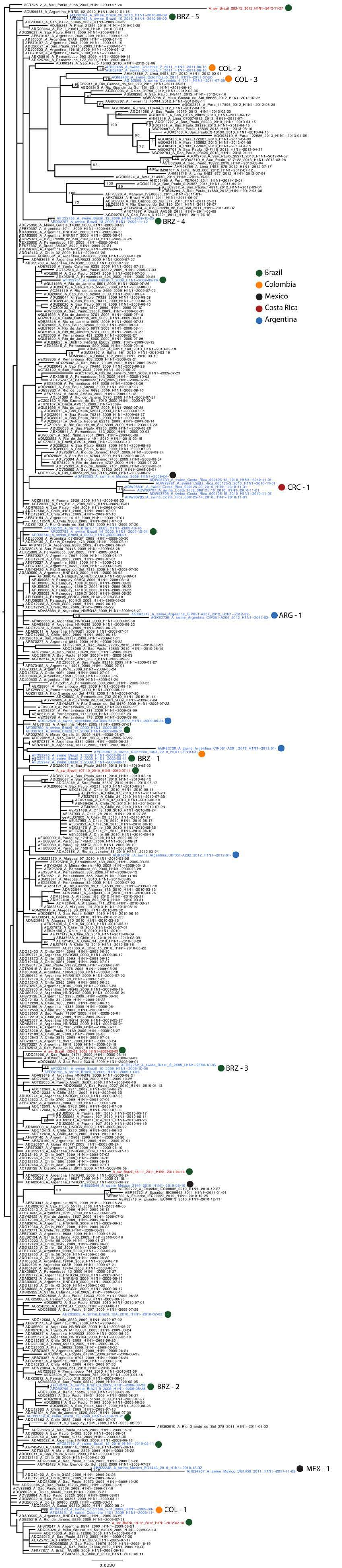

BRZ - 5
COL - 2
COL - 3
BRZ - 4
Brazil
Colombia
Mexico
Costa Rica
Argentina
CRC - 1
ARG - 1
BRZ - 1
BRZ - 3
BRZ - 2
MEX - 1
COL - 1
0.0030

Technical Appendix Figure 5. Maximum-likelihood (ML) tree of human and swine pandemic influenza N1 segments. Phylogenetic relationships of 311 pandemic N1 sequences are inferred using ML methods. Branch lengths represent genetic distance and are drawn to scale. Bootstrap values for key nodes >75 are provided. The N1p alignment comprises 5 viruses sequenced for this study from Brazilian swine (A/swine/Brazil/18/2012/H1N1, A/swine/Brazil/66/2011/H1N1, A/swine/Brazil/12A/2010/H1N1, A/swine/Brazil/107/2010/H1N1, and A/swine/Brazil/263/2012/H1N1); 266 human pandemic N1 sequences collected in Latin America during 2009–2013; and 39 closely related N1 swine sequences collected in Latin America. Shading and numbering of swine viruses are as in Technical Appendix Figure 4.

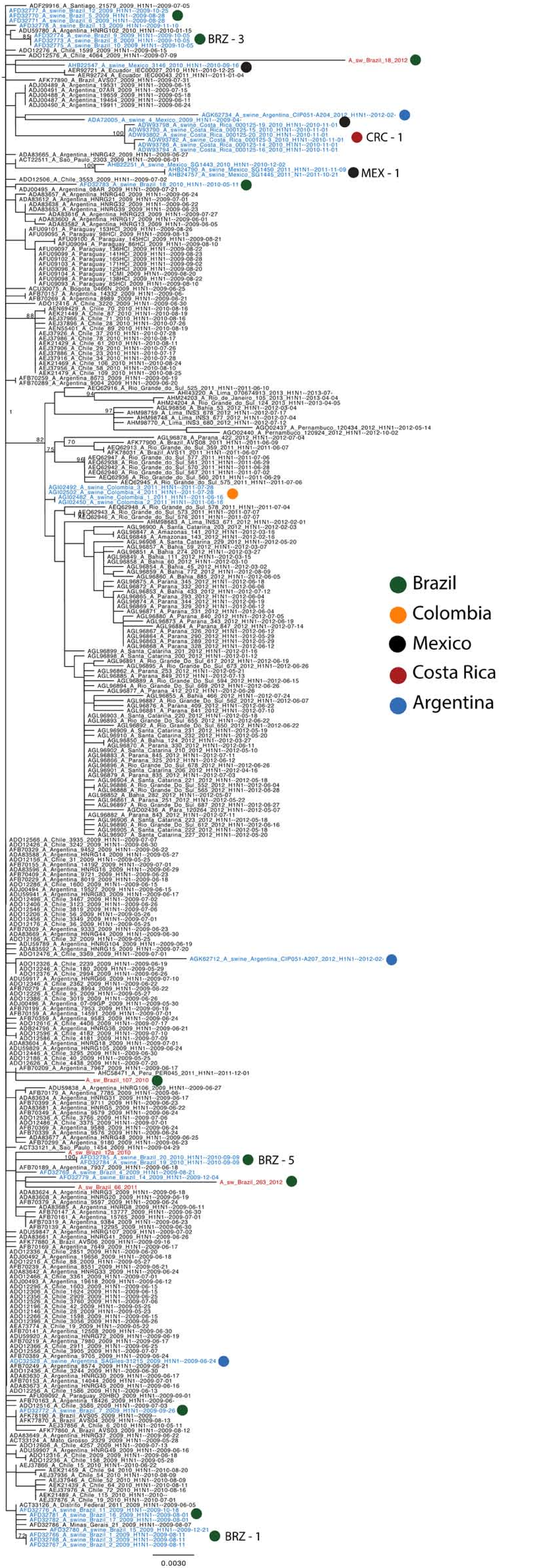
BRZ - 3
CRC - 1
MEX - 1
Brazil
Colombia
Mexico
Costa Rica
Argentina
BRZ - 5
BRZ - 1
0.0030

Technical Appendix Figure 6. Maximum-likelihood (ML) tree of human and swine pandemic influenza polymerase basic (PB) 2 segments. Phylogenetic relationships of 334 pandemic PB2 sequences are inferred using ML methods. Branch lengths represent genetic distance and are drawn to scale. Bootstrap values for key nodes >75 are provided. The PB2 alignment comprises 6 Brazilian swine influenza A viruses sequenced for this study (A/swine/Brazil/66/2011/H1N1, A/swine/Brazil/185-11-7/2011/H1N2, A/swine/Brazil/232-11-13/2011/H1N2, A/swine/Brazil/263/2012/H1N1, A/swine/Brazil/365-11-7/2011/H3N2, A/swine/Brazil/18/2012/H1N1), 4 Brazilian swine influenza A viruses that we previously published (A/swine/Brazil/107/2010/H1N1 [*26*], A/swine/Brazil/31-11-1/2011/H1N2 [*27*], A/swine/Brazil/31-11-3/2011/H1N2 [*27*], A/wild boar/Brazil/214-11-13D/2011/H1N2 [*26*]), 310 human pandemic PB2 sequences collected in Latin America during 2009-2013, and 14 closely related PB2 swine sequences collected in Argentina and Mexico. Shading and numbering of swine viruses are as in Technical Appendix Figure 4.

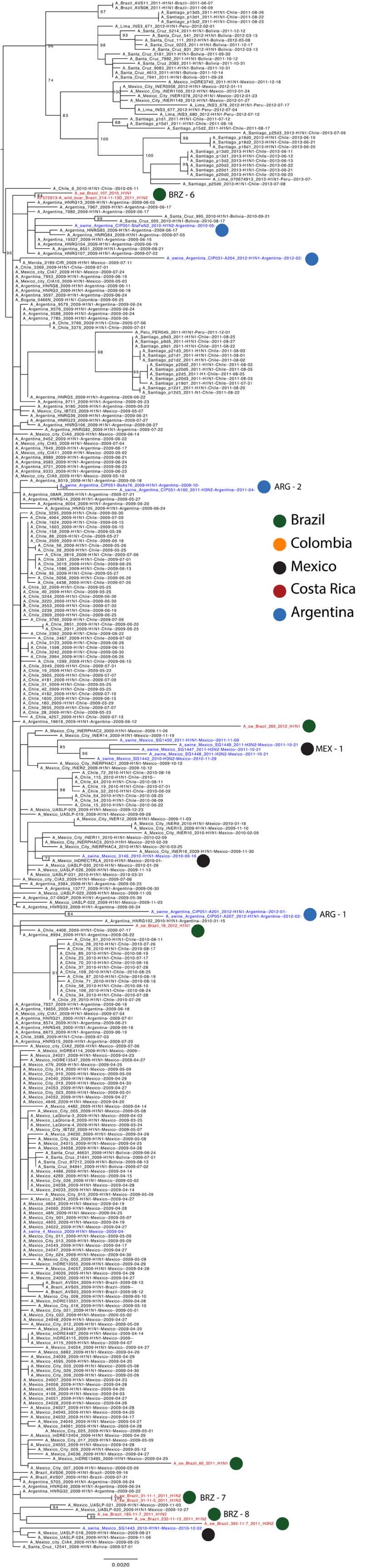

BRZ - 6
ARG - 2
Brazil
Colombia
Mexico
Costa Rica
Argentina
MEX - 1
ARG - 1
BRZ - 7
BRZ - 8
0.0020

Technical Appendix Figure 7. Maximum-likelihood (ML) tree of human and swine pandemic influenza matrix segments. Phylogenetic relationships of 3157 pandemic matrix sequences (10 from swine in Brazil, shaded blue) are inferred using ML methods. Branch lengths represent genetic distance and are drawn to scale. Bootstrap values for key nodes >75 are provided. For clarity, large clades that do not contain Brazilian viruses have been collapsed.

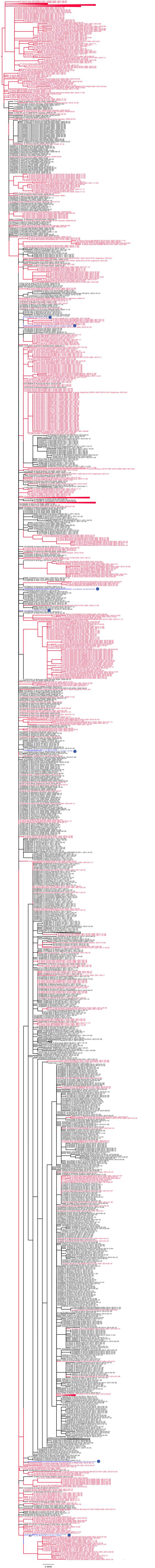

0.0030
